# A New Model Using Routinely Available Clinical Parameters to Predict Significant Liver Fibrosis in Chronic Hepatitis B

**DOI:** 10.1371/journal.pone.0023077

**Published:** 2011-08-11

**Authors:** Wai-Kay Seto, Chun-Fan Lee, Ching-Lung Lai, Philip P. C. Ip, Daniel Yee-Tak Fong, James Fung, Danny Ka-Ho Wong, Man-Fung Yuen

**Affiliations:** 1 Department of Medicine, the University of Hong Kong, Queen Mary Hospital, Hong Kong; 2 Department of Biostatistics, Singapore Clinical Research Institute, Singapore; 3 Center for Quantitative Medicine, Duke-NUS Graduate Medical School, Singapore; 4 Department of Pathology, the University of Hong Kong, Queen Mary Hospital, Hong Kong; 5 Department of Nursing Studies, the University of Hong Kong, Queen Mary Hospital, Hong Kong; Saint Louis University, United States of America

## Abstract

**Objective:**

We developed a predictive model for significant fibrosis in chronic hepatitis B (CHB) based on routinely available clinical parameters.

**Methods:**

237 treatment-naïve CHB patients [58.4% hepatitis B e antigen (HBeAg)-positive] who had undergone liver biopsy were randomly divided into two cohorts: training group (n = 108) and validation group (n = 129). Liver histology was assessed for fibrosis. All common demographics, viral serology, viral load and liver biochemistry were analyzed.

**Results:**

Based on 12 available clinical parameters (age, sex, HBeAg status, HBV DNA, platelet, albumin, bilirubin, ALT, AST, ALP, GGT and AFP), a model to predict significant liver fibrosis (Ishak fibrosis score ≥3) was derived using the five best parameters (age, ALP, AST, AFP and platelet). Using the formula log(index+1) = 0.025+0.0031(age)+0.1483 log(ALP)+0.004 log(AST)+0.0908 log(AFP+1)−0.028 log(platelet), the PAPAS (Platelet/Age/Phosphatase/AFP/AST) index predicts significant fibrosis with an area under the receiving operating characteristics (AUROC) curve of 0.776 [0.797 for patients with ALT <2×upper limit of normal (ULN)] The negative predictive value to exclude significant fibrosis was 88.4%. This predictive power is superior to other non-invasive models using common parameters, including the AST/platelet/GGT/AFP (APGA) index, AST/platelet ratio index (APRI), and the FIB-4 index (AUROC of 0.757, 0.708 and 0.723 respectively). Using the PAPAS index, 67.5% of liver biopsies for patients being considered for treatment with ALT <2×ULN could be avoided.

**Conclusion:**

The PAPAS index can predict and exclude significant fibrosis, and may reduce the need for liver biopsy in CHB patients.

## Introduction

Up to 40% of patients with chronic hepatitis B (CHB) would develop cirrhotic complications or hepatocellular carcinoma (HCC) during their lifetime [1]. While several clinical parameters, including male gender, older age, higher levels of alanine aminotransferase (ALT) and serum HBV DNA have been identified as risk factors for severe liver disease [2], [3], [4], the golden standard in assessing disease severity remains to be liver biopsy. Liver biopsy is still recommended for certain CHB patients, especially those with an ALT level of <2×upper limit of normal (ULN) [5], [6]. However, up to 2% of patients develop complications from liver biopsy [7], [8]. Others problems like intra-observer variation and sampling error are also unavoidable [9], [10], [11]. There is thus an increasing demand for developing predictive models of fibrosis based on non-invasive markers.

Many predictive models of fibrosis, including the AST/platelet radio index (APRI) and FIB-4 index, were based on patients with chronic hepatitis C [12], [13], [14], [15], [16], [17]. Using such models to predict liver fibrosis in CHB patients had produced conflicting results [18], [19]. Only a minority of models were based on CHB patients [20], [21], [22], [23], and these models were limited by a disproportionate percentage of either hepatitis B e antigen (HBeAg)-positive or –negative patients. Some of these studies also lack patients with normal serum ALT [20], [21]. A recently-derived model is the aspartate aminotransferase (AST)/platelet/gamma-glutamyl transpeptidase (GGT)/α-fetoprotein (AFP) (APGA) index, but this is limited by its correlation with transient elastography and not actual liver histology [24]. Another factor limiting the use of other non-invasive models is that markers used in prediction may not be routinely available in non-research laboratories [18], [20], [25], [26].

The aim of this study is to create a predictive model based on routinely-available clinical parameters to accurately predict significant fibrosis in both HBeAg-positive and -negative CHB.

## Methods

### Patients

The current study included treatment-naïve patients who were enrolled into therapeutic drug trials between 1994 and 2008 in the Department of Medicine, the University of Hong Kong, Queen Mary Hospital. All patients were positive for hepatitis B surface antigen (HBsAg) for at least 6 months, with a HBV DNA level of more than 2,000 IU/mL, and a serum ALT of less than 10 times the ULN prior to recruitment. Patients with decompensated cirrhosis or concomitant liver disease, including chronic hepatitis C or D virus infection, primary biliary cirrhosis, autoimmune hepatitis, Wilson's disease, and significant intake of alcohol (20 grams per day for female, 30 grams per day for male) were excluded. Written consent was obtained prior to liver biopsy, and all trials had been approved by the Institutional Review Board of the University of Hong Kong.

Patient demographics and laboratory parameters (altogether 12 variables) were recorded at the time of liver biopsy. These include age, gender, HBeAg status, HBV DNA levels, albumin, bilirubin, ALT, AST, alkaline phosphatase (ALP), GGT, AFP and platelet count. The ULN of ALT was based on the respective drug trial, ranging from 45 to 53 U/L in men and 31 to 43 U/L in women. Serum HBV DNA levels were measured by three different assays, as follow: a branched DNA assay (Versant HBV DNA 3.0 assay, Bayer Health-Care Diagnostic Division, Tarrytown, NY), with a lower limit of quantification of 400 IU/mL in 33 patients, Cobas Amplicor HBV Monitor Test (Roche Diagnostic, Branchburg, NJ) with a lower limit of quantification of 60 IU/mL in 88 patients, and Cobas Taqman assay (Roche Diagnostic, Branchburg, NJ) with a lower limit of quantification of 12 IU/mL in 116 patients.

### Liver Biopsy

An 18G sheathed cutting needle (Temno Evolution, Cardinal Health, McGaw Park, IL) was used for liver biopsy for 33 patients, with a minimum length of 1.5 cm obtained. For the remainder of the cohort, a 17G core aspiration needle (Hepafix, B. Braun Melsungen AG, Germany) was used, with a minimum length of 2 cm obtained. Histologic grading of necroinflammation and staging of liver fibrosis were performed using the Knodell histologic activity index [27] and Ishak fibrosis score [28] respectively, by a single histopathologist blinded to the patients' laboratory data. Significant fibrosis was defined as an Ishak score of 3 or more, meaning the presence of at least bridging fibrosis.

### Statistical analysis

The primary endpoint of the present study was to determine whether there were associations between significant fibrosis which were present in 77 patients (32.4%) in the entire cohort, and the 12 routinely-available clinical parameters mentioned above. Data was randomly divided into a training cohort and a validation cohort. Concerning the optimal sample size of this study, with 32.4% of our patient cohort having significant fibrosis and allowing a 10% error for a 95% confidence interval, 84 patients were needed in each cohort for the study to be adequately powered. A training cohort consisting of 108 patients (45.6%) was used to develop the model. The remaining 129 patients (54.4%) formed the validation cohort. All statistical analyses were performed using SPSS version 16.0 (SPSS Inc., Chicago, IL), SAS system version 9.1, R version 2.81 and STATA/SE 9.2.

To create a new predictive model, all variables were subjected to a logarithmic transformation for a better model fit. The sequence of variables in order of their associations with significant liver fibrosis (co-efficient path) was determined by L1 regularized regression. The area under the receiving operating characteristics (AUROC) curve was determined for each number of variables used for the prediction of significant fibrosis. The number of variables used was decided when the addition of extra variables failed to give a relatively better accuracy. A new predictive model was then created with the optimal cut-off value determined as the value with the highest sensitivity and specificity. Using the new regression model, the AUROC, sensitivity, specificity, positive and negative predictive values and likelihood ratios were calculated.

This new predictive model was compared to three pre-existing non-invasive indexes using routinely-available clinical parameters: the APRI, the FIB-4 index and the APGA index. The APRI was calculated using [AST (U/L)/(ULN of AST)/platelet count (×10^9^/L)]×100 [12]. The FIB-4 index was calculated using [age (years)×AST (U/L)]/{[PLT (10^9^/L)]×(ALT(U/L)]^1/2^}f [13]. The APGA index was calculated using log(index) = 1.44+0.1490log[GGT (U/L)]+0.3308log [AST (U/L)]−0.5846log [platelet count (×10^9^/L)]+0.1148log [AFP (ng/mL)+1] [24].

The Mann-Whitney U test was used for continuous variables with a skewed distribution; the chi-squared test was used for categorical variables. Correlation between different predictive models with significant fibrosis was performed using Spearman correlation co-efficient. A two-sided *p* value of <0.05 was considered statistically significant.

## Results

A total of 237 patients with all 12 clinical parameters available were recruited. The characteristics of all 237 patients at the time of liver biopsy, including a comparison between the training and validation cohorts, are shown in Table 1. The median age was 38.2 years and 98 patients (41.3%) were HBeAg-positive. Twenty-five patients (10.5%) had a normal ALT level. Significant fibrosis and cirrhosis were present in 77 patients (32.4%) and 5 patients (2.1%) respectively. The percentage of patients with significant fibrosis in patients with ALT ≥2×ULN and <2×ULN were 39.6% (44 out of 111 patients) and 26.2% (33 out of 126 patients) respectively.

**Table 1:** Characteristics of 237 patients included in model.

	Total	Training	Validation	*p* value
**Number of patients**	**237**	**108**	**129**	
**Age (years)**	38.2 (18–63)	36.4 (18–63)	40.0 (18–61)	0.695
**Number of male patients**	160 (67.2%)	73 (67.6%)	87 (67.4%)	0.980
**Number of HBeAg-positive patients**	98 (41.3%)	42 (38.9%)	56 (43.4%)	0.481
**Albumin (g/L)**	46 (36–54)	46 (37–54)	45 (36–53)	0.156
**Bilirubin (umol/L)**	12 (3–96)	12 (3–96)	12 (3–31)	0.348
**ALP (U/L)**	76 (20–242)	73.5 (33–145)	76 (20–242)	0.283
**AST (U/L)**	54 (16–304)	52 (16–304)	55 (18–304)	0.490
**ALT (U/L)**	87 (14–507)	80.5 (15–469)	95 (14–507)	0.334
**Number of patients with**				
**• Normal ALT**	25 (10.5%)	10 (9.3%)	15 (11.6%)	
**• ALT 1–2×ULN**	101 (42.6%)	46 (42.6%)	55 (42.6%)	
**• ALT >2×ULN**	111 (46.8%)	52 (48.1%)	59 (45.7%)	
**GGT (U/L)**	33 (5–160)	30.5 (6–134)	35 (5–160)	0.999
**AFP (ng/mL)**	4 (1–178)	4 (1–178)	4 (1–86)	0.420
**Platelet (×10^9^/L)**	201 (93–334)	206.5 (95–331)	198 (93–334)	0.571
**HBV DNA (log IU/mL)**	6.77 (2.70–14.0)	6.99 (3.50–11.8)	6.76 (2.70–14.0)	0.148
**Number of patients with significant necroinflammation (NI≥7)**	120 (50.6%)	47 (43.5%)	73 (56.6%)	0.339
**Number of patients with significant fibrosis (F≥3)**	77 (32.4%)	30 (27.8%)	47 (36.4%)	0.091
**• F6**	5 (2.1%)	3 (2.8%)	2 (1.6%)	
**• F5**	15 (6.3%)	7 (6.5%)	8 (6.2%)	
**• F4**	25 (10.5%)	13 (12.0%)	12 (9.3%)	
**• F3**	32 (13.5%)	7 (6.5%)	25 (19.4%)	
**• F2**	59 (24.8%)	28 (25.9%)	31 (24.0%)	
**• F1**	71 (29.8%)	32 (29.6%)	39 (30.2%)	
**• F0**	30 (12.6%)	18 (16.7%)	12 (9.3%)	

Continuous variables expressed in median (range) F = Ishak Fibrosis Score.

**Figure 1:**
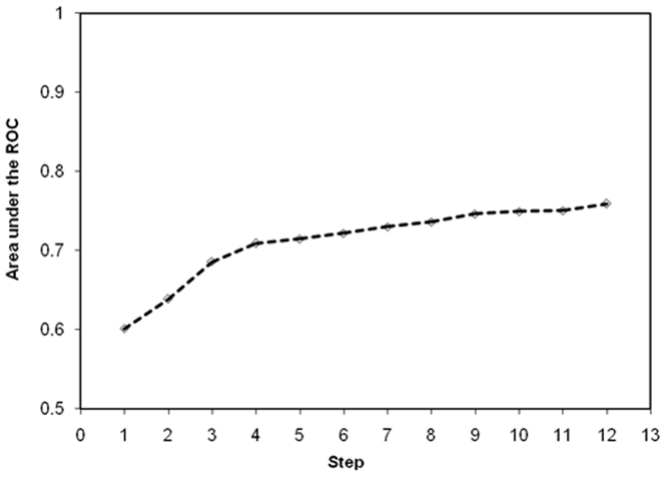
Area under the receiver operating characteristics (AUROC) curve at each step. Steps 1–12 as listed in their order: AFP, ALP, age, AST, platelet count, albumin, HBV DNA, GGT, gender, bilirubin, HBeAg status, ALT.

**Figure 2:**
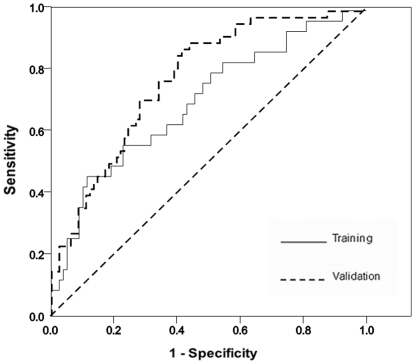
Comparison of receiver operating characteristics (ROC) curves of training and validation cohorts in predicting significant fibrosis for the PAPAS index.

**Figure 3:**
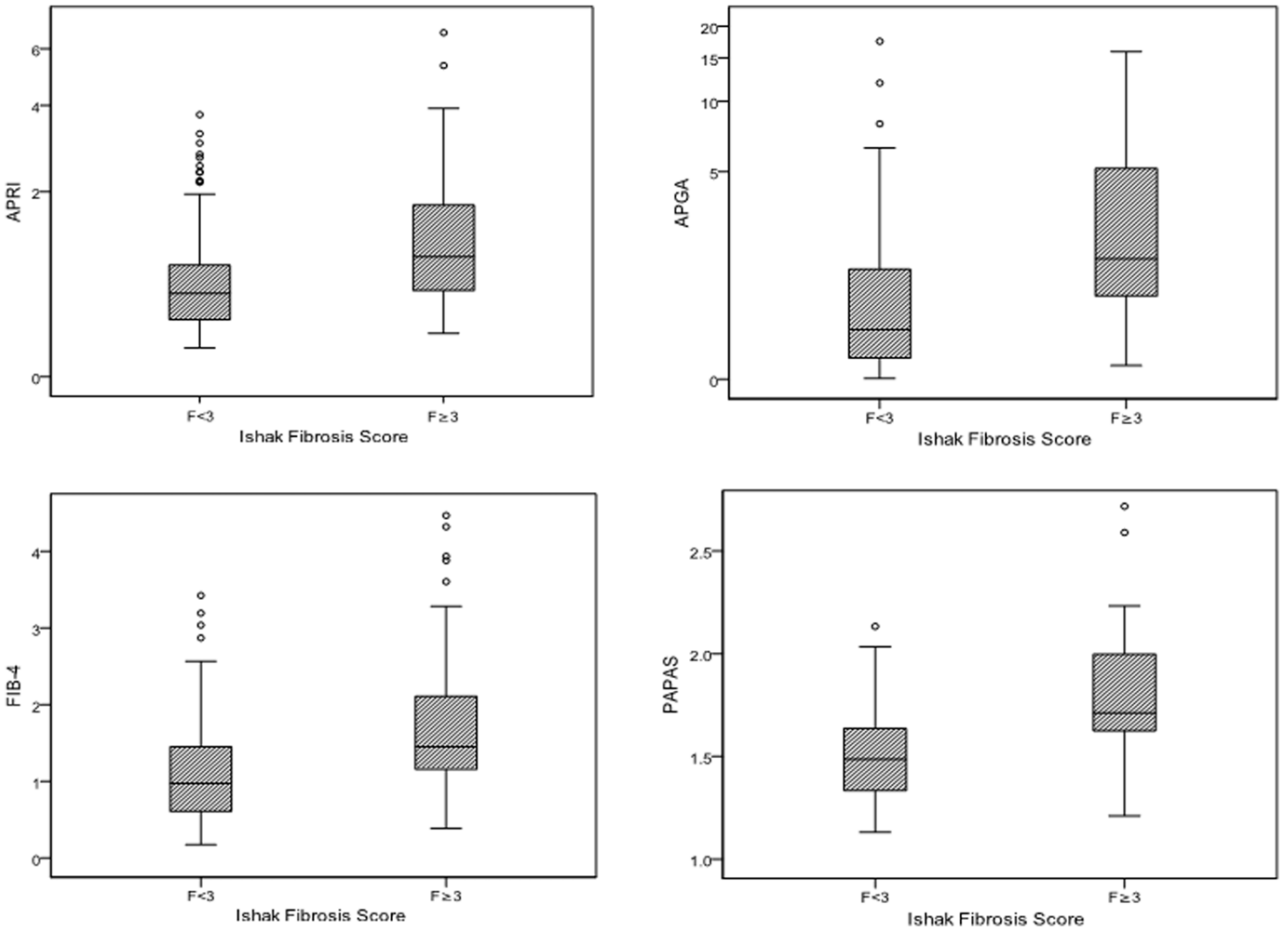
Model values based on Ishak fibrosis score. The top and bottom of each box represents the 25^th^ and 75^th^ percentile interval, the line through the box in the median and the error bars are the 5^th^ and 95^th^ percentile intervals.

**Figure 4:**
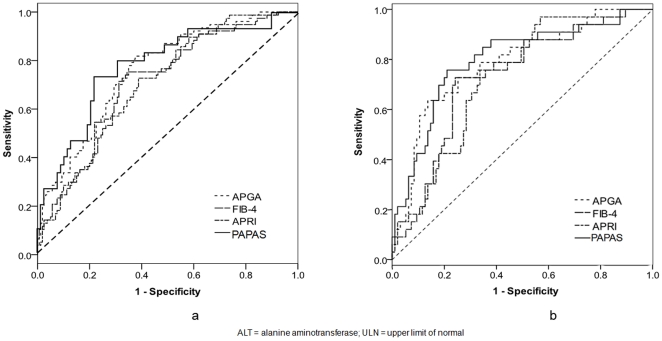
Comparison of ROC curves of different predictive models in predicting significant fibrosis for (a) all patients and (b) patients with ALT <×2 ULN.

The sequence of variables added at each step under the AUROC curve is shown in Figure 1. The addition of the first 5 variables (AFP, ALP, age, AST, platelet count) achieved a best fit in the regression model. The further addition of variables only increases the complexity of the formula without achieving a marked improvement in prediction accuracy. Using L1 regularized regression, a new predictive model for significant fibrosis, named the PAPAS index (Platelet/Age/Phosphatase/AFP/AST), was derived as follows:
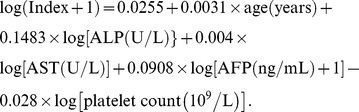



The AUROC for predicting significant fibrosis was 0.701 for the training cohort and 0.776 for the validation cohort (Figure 2). There was no significant difference in the AUCs of both training and validation groups (p = 0.270). The PAPAS index was then compared with three previously published non-invasive indices i.e. the APRI, the FIB-4 index and the APGA index. The boxplots of the four indices in predicting significant fibrosis are shown in Figure 3. APRI, the FIB-4 index, the APGA index and the PAPAS index all correlated well with significant fibrosis [*r* = 0.337, 0.338, 0.418 and 0.426 respectively (all p<0.001)]. The AUROC for predicting significant fibrosis in the validation cohort for all four models is shown in Figure 4a. The AUC of the PAPAS index, APGA index, FIB-4 index and APRI were 0.776, 0.758, 0.723 and 0.708 respectively (Table 2). The AUC of the PAPAS index was significantly better than APRI (p = 0.009). There were no significant differences between the AUCs of PAPAS index, APGA index and FIB-4 index. For patients with ALT <2×ULN, the AUROC for all for indices is shown in Figure 4b. The AUC of the PAPAS index improved to 0.797 (Table 3). The accuracy and correlation coefficients of the PAPAS index are the best among the 4 models.

**Table 2:** Area under curve (AUC) of the validation cohort using the PAPAS index, APGA index, FIB-4 index and APRI for significant fibrosis in all patients.

	AUC for significant fibrosis	95% confidence intervals
**PAPAS**	0.776	0.694–0.854
**APGA**	0.757	0.674–0.840
**FIB-4**	0.723	0.635–0.810
**APRI**	0.708	0.625–0.800

**Table 3:** Area under curve (AUC) of the validation cohort using the PAPAS index, APGA index, FIB-4 index and APRI for significant fibrosis in patients with ALT <2×ULN.

	AUC for significant fibrosis	95% confidence intervals
**PAPAS**	0.797	0.706–0.888
**APGA**	0.784	0.693–0.875
**FIB-4**	0.726	0.629–0.823
**APRI**	0.727	0.636–0.818

The sensitivity, specificity, predictive values and likelihood ratios of all four indices are shown in Table 4, using the various cut-offs suggested for each model. Using an optimal cut-off of 1.662, the PAPAS index had a sensitivity of 73.3% and a specificity of 78.2% in predicting significant fibrosis. The negative predictive value was 88.4%.

**Table 4:** Sensitivity, specificity, predictive values and likelihood ratios of scores according to different cut-offs for predicting significant fibrosis.

	Optimal cut-off	Sensitivity	Specificity	PPV	NPV	LR+	LR−
**PAPAS**	1.662	73.3%	78.2%	56.4%	88.4%	3.365	0.341
**APGA**	6.687	16.9%	98.1%	81.3%	71.0%	9.027	0.847
**FIB-4**	1.45	51.9%	74.4%	49.4%	76.3%	2.028	0.646
	3.25	9.09%	99.4%	87.5%	69.4%	14.670	0.915
**APRI**	0.5	89.6%	40.6%	42.1%	89.0%	1.509	0.256
	1.5	29.9%	88.1%	54.8%	72.3%	2.516	0.796

PPV = positive predictive value.

NPV = negative predictive value.

LR+ = positive likelihood ratio.

LR− = negative likelihood ratio.

One hundred and twenty-six patients (53.2%) among our total patient cohort had an ALT level of <2×ULN, a patient group in whom liver biopsies are recommended before considering treatment. Among this group, 85 patients (67.5%) had a score less than the optimal cut-off of 1.662, suggesting that these patients do not have significant fibrosis and liver biopsies could be avoided. Seventy-five out of these 85 patients (88.2%) had insignificant fibrosis (Ishak stage 0 to 2) on actual histology. For the remaining 10 patients (11.2%), 5 had stage 3 fibrosis and 5 had stage 4 fibrosis. If the revised ULN of ALT as suggested by Prati et al (30 U/L for men, 19 U/L for women) [29] was used, 39 patients would have an ALT level of <2×ULN, of which 30 patients (76.9%) could avoid liver biopsy by having a score of less than 1.662. Twenty-eight out of these 30 patients (93.3%) had insignificant fibrosis. For the remaining 2 patients (6.7%), one had stage 3 fibrosis and another had stage 4 fibrosis.

## Discussion

Given the invasiveness of liver biopsy, the development of non-invasive markers for liver fibrosis has always been an attractive option, especially since non-invasive markers for fibrosis in CHB are not well-established. Liver biopsy itself also has its limitations, thus using the AUROC in evaluating non-invasive markers of fibrosis could never reach the perfect value of 1.0. In fact, it had been shown previously that a perfect marker for significant fibrosis would not even reach an AUROC of 0.90 [30], [31], which is the reason for many previous studies can only obtain an AUROC range of 0.76–0.88 [30].

The PAPAS index obtained an AUROC of 0.776 for the prediction of significant fibrosis. The AUROC improves to 0.797 for patients with ALT <2×ULN, the group of patients with liver biopsy recommended before considering treatment. The sensitivity and specificity of our model were both equally high at 73.3% and 78.2% respectively, and a high negative predictive value of 88.4% was achieved at the optimal cut-off value. The AUROC obtained was superior to other models of fibrosis based on commonly-available clinical parameters used in our cohort. Two such models, the FIB-4 index and APRI, were initially created based on patients with chronic hepatitis C, and therefore might not be suitable for CHB patients. According to one study, the AUROC of APRI in 218 CHB patients in predicting fibrosis was only 0.63 [19]. Two other such models based on chronic hepatitis C patients, Fibrotest and Actitest, achieved satisfactory results in CHB patients, but were limited by the requirement of using special and non-routinely available biomarkers. In addition, the majority of the study population was HBeAg-negative [18]. The disproportionate representation of either HBeAg-positive or HBeAg-negative patients was also seen in other non-invasive models for CHB [20], [21], [22]. Our study had a good mixture of both HBeAg-positive (41.3%) and -negative patients, making it more representative of the whole spectrum of CHB population. Our study also had patients with different ALT ranges, including a proportion of patients with normal ALT.

A high negative predictive value meant the predictive model would excel in excluding CHB patients with significant fibrosis. For patients with an ALT level of <2×ULN, 67.5% of our cohort would be able to avoid the invasiveness of a liver biopsy. Among this subgroup of patients, 88.2% actually had insignificant fibrosis from histology. While 11.8% (10 out of 85) of patients had a discordance between the predictive model score and actual histology, this figure is lower than other studies validating non-invasive models of liver fibrosis [32], [33]. If the revised ULN of ALT as suggested by Prati et al [29] was used, the percentage of patients able to avoid liver biopsy would further increase to 76.9%.

The PAPAS index was based on five common clinical parameters: age, ALP, AST, AFP and platelet count. All 5 parameters had been shown in previous studies to be associated with significant fibrosis in CHB [20], [21], [24]. Age is a valuable predictor since progression of fibrosis in CHB is time-dependent [34], [35]. Increased fibrosis results in a reduced clearance of AST and hence an elevated serum level [36]. A low platelet count has also been associated with advanced liver fibrosis through the altered production of thrombopoietin [37]. The addition of extra variables other than these five parameters did not further improve the accuracy of the current predictive model. Both ALT and HBV DNA levels, known to fluctuate during the natural history of CHB [38], were not included in the PAPAS index. While previous studies had shown several markers, including hyaluronic acid, α-2 macroglobulin and apolipoprotein A_1_, to have a predictive value in CHB, these markers may not be available in the routine evaluation of chronic liver diseases. Using them in predictive models might hinder their widespread use [18], [20], [25], [26].

Many predictive models in previous studies [15], [21], [22], [25] were created using stepwise regression, a prediction method based on identified independent variables to achieve a best-fit model [39]. While commonly used, stepwise regression had been shown to be prone to errors of sampling, measurement and specification [40]. Moreover, a rigid setup in computer programming and a misreading in the order of importance of various predictor variables could result in serious misinterpretation of results [41]. L1 regularized regression adopted in the present study identifies the order in which variables enter or leave the created model, allowing more flexibility in finding a regularized fit with any given number of parameters [42], and has been increasingly used in the design of predictive models in different clinical studies [24], [43], [44], [45].

The current study has certain limitations. Our study only had Chinese CHB patients. Given that 67.6% of patients in our study cohort had limited fibrosis, the study would be biased towards having a high negative predictive value. The PAPAS index was not statistically superior to both the APGA index and FIB-4 index, probably due to the limited number of patients in our present study. Hence, external validation of the PAPAS index with an independent validation cohort would be important before considering widespread use. Body mass index and cholesterol levels were not available in our study, thus we were unable to compare our model with other predictive indices, including the Forns index [15], [21]. Given that the current patient cohort consists of patients with potential to be recruited into drug trials, there would be fewer patients with an inactive disease and low viral load. Our predictive model might not be applicable to this group of patients. However, our cohort included patients with HBV DNA ≥2000 IU/mL, which is the threshold level suggested by CHB guidelines in commencing treatment. Due to the small number of patients with histologic cirrhosis, we were unable to create a predictive model for cirrhosis, which would have less measurement and observer error in detection if possible [11]. Similar to previous models based on CHB patients [20], [21], the PAPAS index did not achieve a high positive predictive value. Therefore, the PAPAS index will be best applicable in excluding patients with insignificant fibrosis in whom treatment may not be necessary at the time of measurement. For patients with the score above the optimal cut-off level of 1.662, the decision of treatment should be considered in conjunction with other disease parameters or viral markers.

A possible method to improve the diagnostic accuracy of predictive models is to combine the available clinical parameters with imaging or transient elastography. The former had been attempted by including the spleen size on imaging, with a high positive predictive value for cirrhosis obtained [23]. The accuracy of transient elastography in CHB is hindered whenever the ALT levels are elevated [46], but this could be improved by combining transient elastography with a non-invasive predictive model like the Forns index [47]. The sequential use of non-invasive markers is also another option [48], although such studies are lacking in CHB patients.

In conclusion, the PAPAS index, a newly-designed predictive model using routinely-available clinical parameters, can accurately predict significant liver fibrosis in CHB patients, and potentially reduce the need for liver biopsies. Further studies would be needed to validate this model and compare it with other non-invasive models of fibrosis in CHB.
